# Acaricidal target and mite indicator as color alteration using 3,7-dimethyl-2,6-octadienal and its derivatives derived from *Melissa officinalis* leaves

**DOI:** 10.1038/s41598-018-26536-9

**Published:** 2018-05-25

**Authors:** Jun-Hwan Park, Hoi-Seon Lee

**Affiliations:** Department of Bioenvironmental Chemistry, Chonbuk National University, Jeonju, 54896 Republic of Korea

## Abstract

Toxicities and color deformation were evaluated of essential oils of *Melissa officinalis* cultivated in France, Ireland, and Serbia and their constituents, along with the control efficacy of spray formulations (0.25, 0.5, and 1%) containing *M*. *officinalis* oils cultivated in France and its main compound against *Dermatophagoides farinae* and *D*. *pteronyssinus* adults. In a contact + fumigant bioassay, *M*. *officinalis* oil (France) was more active against *D*. *farinae* and *D*. *pteronyssinus*, compared to *M*. *officinalis* oils (Ireland and Serbia). Interestingly, color alteration of *D*. *farinae* and *D*. *pteronyssinus* was exhibited, changing from colorless to golden brown through the treatment with *M*. *officinalis* oils. The acaricidal and color alteration principle of three *M*. *officinalis* oils was determined to be 3,7-dimethyl-2,6-octadienal. *M*. *officinalis* oil (France) and 3,7-dimethyl-2,6-octadienal were significantly more effective in closed containers than in open containers, indicating that their acaricidal route of action was largely a result of vapor action. Sprays (0.5 and 1%) containing 3,7-dimethyl-2,6-octadienal and 1% spray containing *M*. *officinalis* oil (France) resulted in 100% mortality and color alteration against *D*. *farinae* and *D*. *pteronyssinus*. These results indicated that *M*. *officinalis* oil and 3,7-dimethyl-2,6-octadienal could be developed as a suitable acaricidal and mite indicator ingredient for the control of dust mites.

## Introduction

House dust mites (HDMs), such as *Dermatophagoides farinae* and *D*. *pteronyssinus*, are an important source of atopic dermatitis, perennial rhinitis, and asthma, with more than a million people at risk in the world^1^. HDMs, mainly inhabit the house, apartment, and workplace^1,2^. Changes in housing lifestyle, such as individual households in flats, together with carpets and central installed heating, have increased the conditions for the multiplication of *D*. *farinae* and *D*. *pteronyssinus*^3^. In addition, HDM allergy is also caused by mite faecal pellets, eggs, and dead mite bodies, making it difficult to control mites from the indoor environment^4^. Control of *D*. *farinae* and *D*. *pteronyssinus* has been conducted by chemical or physical methods^5^. Synthetic acaricides are most commonly used, and include chemical agents containing benzyl benzoate, dibutyl phthalate *N*,*N*-diethyl-*m*-toluamide (DEET) and pyrethroids^6^. Although these acaricides are effective, misuse of synthetic acaricides has led to serious drawbacks, including acaricidal resistance, toxicity of human and animals, and environmental hazards^7^. In recent years, the development of natural insecticides derived from plants and microbes is required to overcome these drawbacks. Plant-derived products (e.g. 2-isopropyl-5-methylphenol and geraniol) that have wide bioactivity against hygienic insect pests and harmful insects have been researched as alternatives to existing insecticides^8,9^.

*Melissa officinalis* L. is a well-known medicinal plant that is widely cultivated throughout Europe^10^. Because it has lemon-like flavor and fragrance, *M*. *officinalis* is commonly referred to as Lemon Balm^11^. *M*. *officinalis* oil was evaluated by antifungal, antibacterial, and antioxidant agents, and is applied to many areas of the cosmetic, food, medicine, and perfume industries^10,12^. The chemical composition of *M*. *officinalis* oils cultivated in different countries has been investigated by many researchers^13,14^. Patora *et al*.^13^ reported that *M*. *officinalis* oil cultivated in Poland is characterized by a high content of β-caryophyllene oxide. Moreover, Basta *et al*.^14^ reported that the major constituent of *M*. *officinalis* oil cultivated in Greece was caryophyllene oxide, while other predominant constituents were (*E*)-caryophyllene, sabinene and β-pinene.

To the best of our knowledge, no reports are available on acaricidal toxicities and the color deformation effects of *M*. *officinalis* oils cultivated in France, Ireland, and Serbia against *D*. *farinae* and *D*. *pteronyssinus*. Therefore, the objective of this study was to determine the chemical composition of the essential oils of *M*. *officinalis* cultivated in France, Ireland, and Serbia, and to evaluate their acaricidal and color deformation properties against *D*. *farinae* and *D*. *pteronyssinus*. We further tested spray formulations containing 0.25, 0.5, and 1% *M*. *officinalis* oil cultivated in France and its main compound, to investigate the most effective formulations for use as a future acaricide and mite indicator.

## Results

### Acaricidal activities and color deformation effects of the essential oils of *M*. *officinalis* cultivated in the three different countries

The acaricidal activities of *M*. *officinalis* oils cultivated in France, Ireland, and Serbia against *D*. *farinae* and *D*. *pteronyssinus* were evaluated with the contact + fumigant bioassay, and compared with that of the synthetic acaricide, *N*,*N*-diethyl-*m*-toluamide (DEET) (Table 1). Based on the LD_50_ values, the most toxic oil against *D*. *farinae* and *D*. *pteronyssinus* was *M*. *officinalis* oil (LD_50_, 3.91 and 3.53 µg/cm^2^) cultivated in France, followed by *M*. *officinalis* oil (LD_50_, 5.29 and 4.97 µg/cm^2^) cultivated in Ireland and *M*. *officinalis* oil (LD_50_, 5.50 and 5.85 µg/cm^2^) cultivated in Serbia. The three types of oils were about 3.6–5.1 and 2.5–4.1 times more toxic than DEET (LD_50_, 19.98 and 14.44 µg/cm^2^) against *D*. *farinae* and *D*. *pteronyssinus*, respectively (Table 1).

**Table 1:** Acaricidal activities of the essential oils of *M*. *officinalis* in France, Ireland, and Serbia and synthetic acaricide against *D*. *farinae* and *D*. *pteronyssinus*. using a contact + fumigant toxicity bioassay.

Geographical origin	Mite species	LD_50_ (μg/cm^2^) (95% CL)^a^	LD_90_ (μg/cm^2^) (95% CL)^a^	Slope ± SE	χ^2^ value (df, *p*)	RT^b^
France	*D*. *farinae*	3.91 (3.23–4.64)	10.71 (8.25–15.19)	2.38 ± 0.33	2.864 (5, 0.721)	5.1
	*D*. *pteronyssinus*	3.53 (2.90–4.11)	9.33 (7.25–13.01)	1.40 ± 0.35	1.693 (5, 0.890)	4.1
Ireland	*D*. *farinae*	5.29 (4.44–6.27)	14.28 (11.13–24.77)	2.63 ± 0.36	1.869 (5, 0.760)	3.8
	*D*. *pteronyssinus*	4.97 (3.67–6.41)	20.57 (15.10–30.61)	2.08 ± 0.27	6.439 (4, 0.169)	2.9
Serbia	*D*. *farinae*	5.50 (4.64–6.51)	14.61 (11.38–21.65)	3.02 ± 0.40	2.785 (5, 0.594)	3.6
	*D*. *pteronyssinus*	5.85 (4.45–7.36)	19.15 (14.70–27.68)	2.49 ± 0.31	3.580 (4, 0.466)	2.5
DEET	*D*. *farinae*	19.98 (17.61–22.77)	37.69 (31.52–49.92)	4.65 ± 0.64	1.132 (4, 0.889)	1.0
	*D*. *pteronyssinus*	14.44 (12.56–16.64)	29.69 (24.33–40.85)	4.29 ± 0.61	4.381 (4, 0.357)	1.0

(^a^LD_50_/LD_90_ is the average of 3 determinations, with 30 adult mites per replication. ^b^RT_50_, Relative toxicity = LD_50_ value of DEET/LD_50_ value of each compound; Exposed for 24 h).

**Figure 1:**
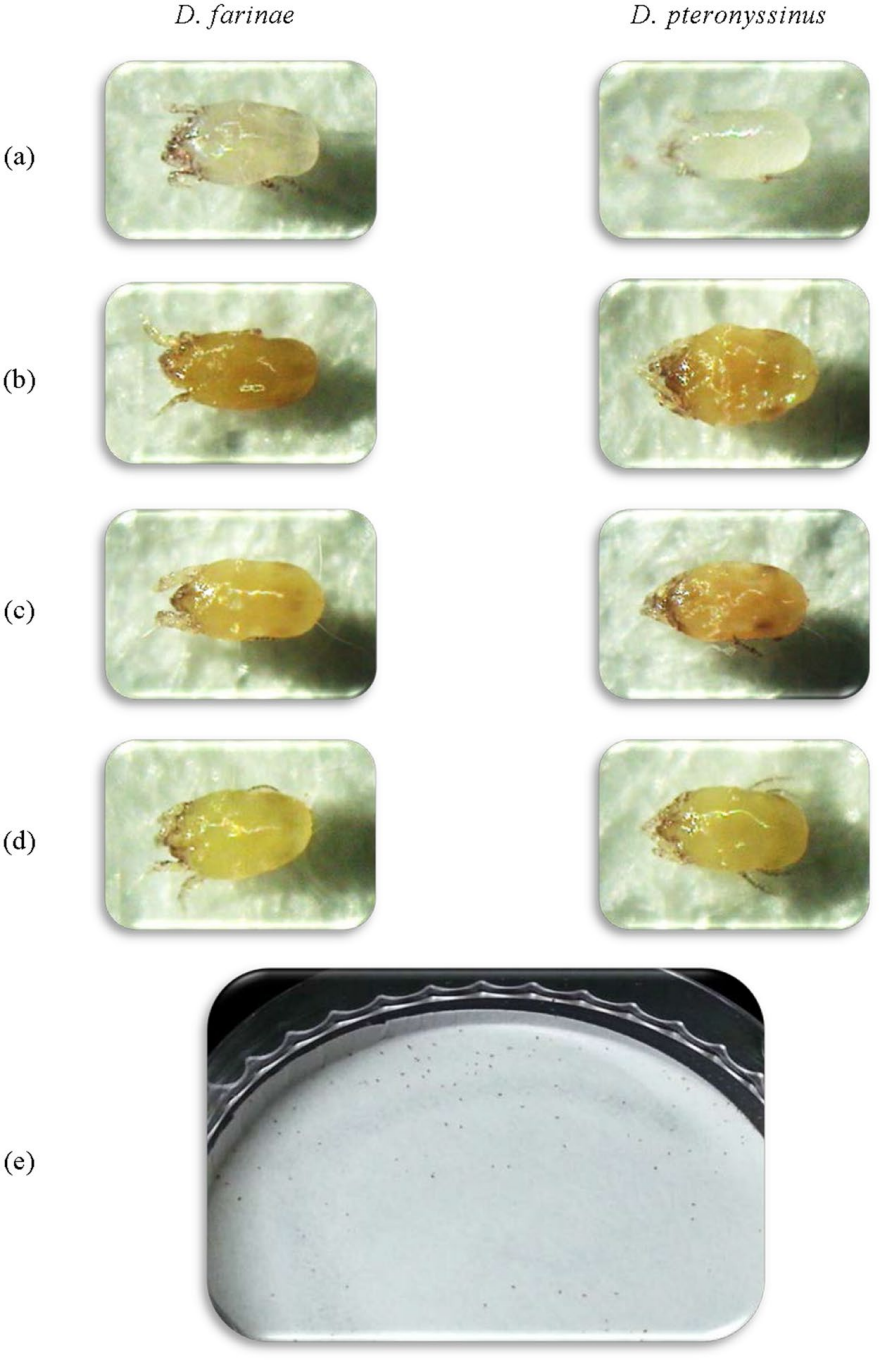
Color deformation effects of the three oils of *M*. *officinalis* cultivated in France, Ireland, and Serbia against American house dust mites and European house dust mite. (**a**) untreated mites, (**b**) mites, treated with *M*. *officinalis* oil (France), (**c**) mites, treated with *M*. *officinalis* oil (Ireland), (**d**) mites, treated with *M*. *officinalis* oil (Serbia) (100×), and (**e**) mites, treated with *M*. *officinalis* oil (France) (naked eye).

The color deformation effects of *M*. *officinalis* oils cultivated in France, Ireland, and Serbia against *D*. *farinae* and *D*. *pteronyssinus* were investigated using the contact + fumigant bioassay (Fig. 1). After 24 h of treatment with two-fold of the contact + fumigant LD_90_ values of each sample, there was significant difference in color alteration between treated mites with each sample and untreated mites. As a result, while the untreated mites (*D*. *farinae* and *D*. *pteronyssinus*) were colorless (Fig. 1(a)), the mites treated with *M*. *officinalis* oils cultivated in France, Ireland, and Serbia presented with color alteration to a golden brown color of their body (Fig. 1(b–d)), and the color alterations that were visible with the naked eye instead of light microscope were confirmed by Fig. 1(e).

### Chemical composition of *M*. *officinalis* oils cultivated in the three different countries

To further explore the acaricidal toxicities and color deformation effects of *M*. *officinalis* oils cultivated in France, Ireland, and Serbia against *D*. *farinae* and *D*. *pteronyssinus*, the components of the essential oils of *M*. *officinalis* cultivated in France, Ireland, and Serbia were investigated by GC-MS analysis. Supplementary Table S1 shows the main components of each oil. A total of 13 compounds were identified in the essential oil of *M*. *officinalis* cultivated in France, which represented 97.67% (area percent) of the oil extracted. The main compounds were 3,7-dimethyl-2,6-octadienal at 43.37%, β-caryophyllene (27.42%), and germacrene D (14.46%). From the essential oil of *M*. *officinalis* cultivated in Ireland, 11 components were identified, representing 96.09% of the total oil, among which β-caryophyllene (30.75%), 3,7-dimethyl-2,6-octadienal (26.90%), β-cubebene (11.19%), 3,7-dimethyl-6-octenal (8.65%), and δ-cadinene (7.97%) were the main compounds. The essential oil of *M*. *officinalis* cultivated in Serbia yielded 11 compounds that represented 97.82% of the total oil, with β-caryophyllene (32.71%), 3,7-dimethyl-2,6-octadienal (21.88%), β-cubebene (21.54%), and 3,7-dimethyl-6-octenal (7.64%) being the main compounds. In all cases, the most abundant were sesquiterpene hydrocarbons (46.99–62.94%), followed by monoterpene aldehyde (29.55–45.32%), monoterpene ester (2.45–5.06%), monoterpene hydrocarbon (1.38–2.81%) and monoterpene alcohol (0–1.53%).

### Acaricidal activities and color deformation effects of constituents derived from *M*. *officinalis* oils from the three different countries

In order to identify the major component that is responsible for the contact + fumigant toxicity and color deformation effect, the acaricidal activities and color deformation effects of 7 commercial components (caryophyllene oxide, β-caryophyllene, geranyl acetate, 6-methyl-5-hepten-2-one, 3,7-dimethyl-6-octenal, 3,7-dimethyl-2,6-octadienal, and 3,7-dimethyl-1,3,6-octatrien) of *M*. *officinalis* oils cultivated in France, Ireland, and Serbia were evaluated using contact + fumigant toxicity bioassay against *D*. *farinae* and *D*. *pteronyssinus*, and compared with positive control, DEET (Table 2). Based on the LD_50_ values, the most toxic compound against *D*. *farinae* and *D*. *pteronyssinus* was 3,7-dimethyl-2,6-octadienal (LD_50_, 2.92 and 2.61 µg/cm^2^), followed by 6-methyl-5-hepten-2-one (LD_50_, 4.44 and 5.79 µg/cm^2^), caryophyllene oxide (LD_50_, 5.07 and 6.35 µg/cm^2^), β-caryophyllene (LD_50_, 7.01 and 7.81 µg/cm^2^), 3,7-dimethyl-6-octenal (LD_50_, 7.06 and 8.41 µg/cm^2^) and geranyl acetate (LD_50_, 19.48 and 16.13 µg/cm^2^). In contrast, 3,7-dimethyl-1,3,6-octatrien did not show any acaricidal toxicity against *D*. *farinae* and *D*. *pteronyssinus* in the contact + fumigant toxicity bioassay.

**Table 2:** Acaricidal activities of major components of the essential oils of *M*. *officinalis* cultivated in France, Ireland, and Serbia against *D*. *farinae* and *D*. *pteronyssinus* using a contact + fumigant toxicity bioassay.

Compounds	Species	LD_50_ (μg/cm^2^) (95% CL)^a^	LD_90_ (μg/cm^2^) (95% CL)^a^	Slope ± SE	χ^2^ value (df, *p*)	CDE^b^
Caryophyllene oxide	*D*. *farinae*	5.07 (3.71–6.51)	18.70 (14.07–28.05)	2.26 ± 0.30	5.295 (4, 0.258)	—^d^
	*D*. *pteronyssinus*	6.35 (4.89–7.98)	22.30 (16.72–34.05)	2.35 ± 0.30	4.802 (4, 0.308)	—
β-Caryophyllene	*D*. *farinae*	7.01 (5.17–8.86)	22.52 (17.42–32.53)	2.53 ± 0.35	6.039 (4, 0.196)	—
	*D*. *pteronyssinus*	7.81 (6.01–9.71)	27.38 (20.77–39.32)	2.65 ± 0.31	3.297 (5, 0.509)	—
Geranyl acetate	*D*. *farinae*	19.48 (13.08–26.86)	45.16 (31.47–68.15)	3.51 ± 0.44)	7.936 (4, 0.094)	—
	*D*. *pteronyssinus*	16.13 (13.29–19.44)	39.75 (31.27–45.77)	3.27 ± 0.42	2.199 (4, 0.699)	—
6-Methyl-5-hepten-2-one	*D*. *farinae*	4.44 (3.54–5.47)	15.24 (11.33–24.14)	2.39 ± 0.32	5.399 (4, 0.249)	—
	*D*. *pteronyssinus*	5.79 (4.86–6.90)	16.09 (12.40–24.30)	2.89 ± 0.39	1.812 (4, 0.770)	—
3,7-Dimethyl-6-octenal	*D*. *farinae*	7.06 (5.59–8.65)	22.25 (17.18–32.47)	2.57 ± 0.33	5.052 (5, 0.282)	—
	*D*. *pteronyssinus*	8.41 (6.13–11.18)	29.30 (19.75–47.17)	2.36 ± 0.29	7.439 (4, 0.114)	—
**3,7-Dimethyl-2,6-octadienal**	*D*. *farinae*	2.92 (2.30–3.55)	8.04 (6.34–11.53)	2.43 ± 0.36	14.522 (5, 0.911)	Ο^e^
	*D*. *pteronyssinus*	2.61 (2.05–3.15)	6.71 (5.14–9.93)	2.27 ± 0.37	1.159 (5, 0.949)	Ο
3,7-Dimethyl-1,3,6-octatrien	*D*. *farinae*	—^c^	—	—	—	—
	*D*. *pteronyssinus*	—	—	—	—	—
DEET	*D*. *farinae*	19.98 (17.61–22.77)	37.69 (31.52–49.92)	4.65 ± 0.64	1.132 (4, 0.889)	—
	*D*. *pteronyssinus*	14.44 (12.56–16.64)	29.69 (24.33–40.85)	4.29 ± 0.61	4.381 (4, 0.357)	—

^a^LD_50_/ LD_90_ is the average of 3 determinations, with 30 adult mites per replication; Exposed for 24 h. ^b^Color deformation effect. ^c^No activity. ^d^Not observed, ^e^Observed.

**Figure 2:**
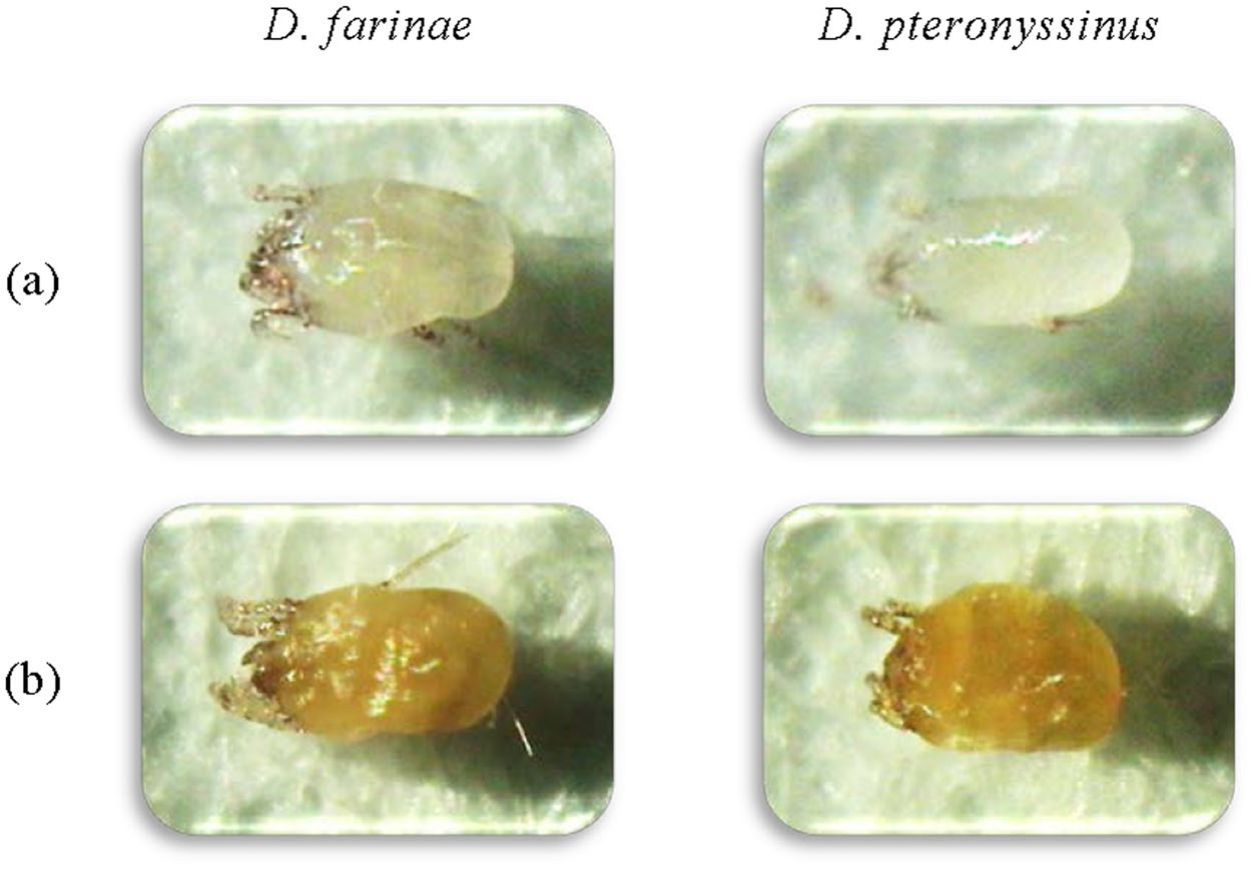
Color deformation effects of 3,7-dimethyl-2,6-octadienal treated mites against American house dust mites and European house dust mites. (**a**) untreated mites, and (**b**) mites, treated with 3,7-dimethyl-2,6-octadienal (100×).

The color deformation effects of 7 constituents derived from *M*. *officinalis* oils cultivated in France, Ireland, and Serbia against *D*. *farinae* and *D*. *pteronyssinus* were investigated using the contact + fumigant bioassay (Fig. 2). After 24 h of treatment with two-fold of the contact + fumigant LD_90_ values of each sample, there was significant difference in the color alteration between mites treated with 3,7-dimethyl-2,6-octadienal and mites treated with caryophyllene oxide, β-caryophyllene, geranyl acetate, 6-methyl-5-hepten-2-one, 3,7-dimethyl-6-octenal, or 3,7-dimethyl-1,3,6-octatrien. The body of the treated mites with 3,7-dimethyl-2,6-octadienal became golden brown color (Fig. 2), whereas the treated mites with the others were colorless.

### Route of acaricidal action of *M*. *officinalis* oil cultivated in France and 3,7-dimethyl-2,6-octadienal

To determine whether the acaricidal activities of *M*. *officinalis* oil cultivated in France and 3,7-dimethyl-2,6-octadienal against *D*. *farinae* and *D*. *pteronyssinus* were attributable to contact or fumigant action, the vapor phase toxicity bioassay in two formats (closed and open container) was used (Table 3). After 24 h of exposure to 21.42 µg/cm^3^ of *M*. *officinalis* oil (France), there was a significant difference in lethal activity (*P* < 0.0001) between exposure in a closed container, which resulted in 100% mortality, and exposure in an open container, which resulted in 8% mortality against *D*. *farinae*. Similar differences in the response of *D*. *farinae* to 3,7-dimethyl-2,6-octadienal (exposure to 16.08 µg/cm^3^) in closed (100% mortality) and open container (12% mortality) treatments were observed. Against *D*. *pteronyssinus*, there was a significant difference (P < 0.0001) in the acaricidal toxicity of 18.66 µg/cm^3^ of *M*. *officinalis* oil (France) between closed (100% mortality) and open containers (10% mortality) (Table 3). Similar differences in the response of *D*. *pteronyssinus* to 3,7-dimethyl-2,6-octadienal (exposure to 13.42 µg/cm^3^) in closed (100% mortality) and open container (16% mortality) were observed. No mortality was observed in the positive control (only ethanol) exposure, in both formats.

**Table 3:** Route of acaricidal action of *M*. *officinalis* oil (France) and 3,7-dimethyl-2,6-octadienal against *D*. *farinae* and *D*. *pteronyssinus* using a vapor-phase toxicity bioassay.

Samples	Species	Concentration (μg/cm^3^)^a^	Mortality (%) ( ± SE)^b^		*P*-value^c^
			Vapor in closed container	Vapor in open container	
*M*. *officinalis* oil (France)	*D*. *farinae*	21.42	100 (±0.0)	8 (±1.4)	0.0001
	*D*. *pteronyssinus*	18.66	100 (±0.0)	10 (±1.2)	0.0001
3,7-Dimethyl-2,6-octadienal	*D*. *farinae*	16.08	100 (±0.0)	12 (±1.6)	0.0001
	*D*. *pteronyssinus*	13.42	100 (±0.0)	16 (±2.1)	0.0001

^a^Treatment with ca. two-fold LD_90_ value of each sample; Exposed for 24 h. ^b^Each experiment was performed 3 times and the data averaged. ^c^According to Student’s *t*-test.

### Effectiveness of the essential oil of *M*. *officinalis* cultivated in France and 3,7-dimethyl-2,6-octadienal against *Dermatophagoides* spp. covered with three thicknesses of non-woven fabric

The lethal activities of *M*. *officinalis* oil (France) and 3,7-dimethyl-2,6-octadienal against *D*. *farinae* and *D*. *pteronyssinus* covered with three thicknesses (1.0, 2.5, and 3.0 mm) of non-woven fabric were evaluated using fumigant chamber (Supplementary Fig. S1) (Table 4). *M*. *officinalis* oil (France) at dose of 21.42 µg/cm^3^ caused 100% mortality against *D*. *farinae* covered with 1.0, 2.5, or 3.0 mm thickness non-woven fabric, respectively; and at dose of 18.66 µg/cm^3^, also caused 100% mortality against *D*. *pteronyssinus* covered with 1.0, 2.5, or 3.0 mm thickness non-woven fabric, respectively, within 24 h. Furthermore, 3,7-dimethyl-2,6-octadienal at dose of 16.08 µg/cm^3^ caused 100% mortality to *D*. *farinae* covered with 1.0, 2.5, or 3.0 mm thickness non-woven fabric, respectively, and at dose of 13.42 µg/cm^3^, also caused 100% mortality against *D*. *pteronyssinus* covered with 1.0, 2.5, or 3.0 mm thickness non-woven fabric, respectively, within 24 h (Table 4).

**Table 4:** Fumigant toxicity of *M*. *officinalis* oil (France) and 3,7-dimethyl-2,6-octadienal against *D*. *farinae* and *D*. *pteronyssinus* covered with different thicknesses of non-woven fabric.

ample^a^	Thickness (mm)	Mortality (%) (±SE) over time^b^							
		*Dermatophagoides farinae*				*Dermatophagoides pteronyssinus*			
		2 h	6 h	12 h	24 h	2 h	6 h	12 h	24 h
*M*. *officinalis* oil (France)	1.0	23 (±1.9) a	52 (±3.2) a	84 (±2.6) a	100 (±0.0)	28 (±4.4) a	67 (±5.5) a	87 (±2.0) a	100 (±0.0)
	2.5	11 (±2.2) b	36 (±2.9) b	68 (±3.1) b	100 (±0.0)	8 (±1.5) b	41 (±2.3) b	72 (±4.0) b	100 (±0.0)
	3.0	6 (±2.0) b	32 (±1.2) b	64 (±4.6) b	100 (±0.0)	4 (±1.2) b	34 (±2.9) b	62 (±2.6) c	100 (±0.0)
3,7-Dimethyl-2,6-octadienal	1.0	33 (±2.6) a	55 (±3.0) a	82 (±2.3) a	100 (±0.0)	38 (±2.3) a	57 (±2.1) a	86 (±3.8) a	100 (±0.0)
	2.5	13 (±1.7) b	38 (±3.6) b	72 (±2.1) b	100 (±0.0)	16 (±3.2) b	42 (±1.8) b	76 (±3.5) b	100 (±0.0)
	3.0	4 (±0.3) c	32 (±4.3) b	62 (±1.7) c	100 (±0.0)	12 (±2.7) b	38 (±2.3) b	60 (±5.3) c	100 (±0.0)

^a^Treatment with ca. two-fold LD_90_ value of each sample; Exposed for 24 h. ^b^Each experiment was performed 3 times and the data averaged; Means within a column followed by the same letter are not significantly different (P = 0.05, Scheffé test).

### Effectiveness of *M*. *officinalis* oil (France) and 3,7-dimethyl-2,6-octadienal applied as sprays

The control efficacy of *M*. *officinalis* oil (France) spray formulations (MO-0.25%, MO-0.5%, MO-1.0%), 3,7-dimethyl-2,6-octadienal spray formulations (DO-0.25%, DO-0.5%, DO-1.0%), and commercial permethrin spray formulation (2.5 g/L) were investigated using direct and indirect application methods against *D*. *farinae* and *D*. *pteronyssinus* (Table 5). In the direct and indirect application methods, 1% sprays containing *M*. *officinalis* oil (France) (MO-1.0) resulted in 100% mortality against *D*. *farinae* and *D*. *pteronyssinus*, whereas the 0.5% sprays (MO-0.5) resulted in >80% mortality against *D*. *farinae* and *D*. *pteronyssinus*. The lethalities of the 0.25% spray containing *M*. *officinalis* oil (France) (MO-0.25) in direct and indirect application methods were 68 and 55%, and 62 and 68% against *D*. *farinae* and *D*. *pteronyssinus*, respectively. 3,7-Dimethyl-2,6-octadienal applied as 1 and 0.5% spray (DO-1 and DO-0.5) in direct and indirect applications provided 100% mortality of *D*. *farinae* and *D*. *pteronyssinus*, whereas the 0.25% (DO-0.25) resulted in 84 and 72%, and 88 and 78% mortality against *D*. *farinae* and *D*. *pteronyssinus*, respectively. Permethrin spray treatment in direct and indirect application resulted in 18 and 14%, and 21 and 25% mortality against *D*. *farinae* and *D*. *pteronyssinus*, respectively. DEET applied as 1% spray (DEET-1) in direct and indirect application methods provided 8 and 0%, and 12 and 0% mortality against *D*. *farinae* and *D*. *pteronyssinus*, respectively. There was no significant difference in toxicity of the three spray formulations between the direct and indirect application methods. There was no mortality for the negative control (ethanol–castor oil–water) treated mites in the direct and indirect spray application methods.

**Table 5:** Effectiveness of three spray formulations containing *M*. *officinalis* oil (France) and 3,7-dimethyl-2,6-octadienal against *D*. *farinae* and *D*. *pteronyssinus* using direct and indirect application methods during a 4 h exposure.

Sample^a^	Mortality (%) ( ± SE)^b^			
	*D*. *farinae*		*D*. *pteronyssinus*	
	A^c^	B^c^	A	B
MO-0.25	68 (±3.1)^c^	58 (±1.8)^c^	62 (±2.7)^c^	68 (±3.5)^b^
MO-0.5	88 (±2.6)^b^	82 (±3.0)^b^	94 (±3.1)^a^	90 (±2.5)^a^
MO-1	100 (±0.0)^a^	100 (±0.0)^a^	100 (±0.0)^a^	100 (±0.0)^a^
DO-0.25	84 (±2.0)^b^	72 (±1.8)^b^	88 (±2.2)^b^	78 (±2.2)^b^
DO-0.5	100 (±0.0)^a^	100 (±0.0)^a^	100 (±0.0)^a^	100 (±0.0)^a^
DO-1	100 (±0.0)^a^	100 (±0.0)^a^	100 (±0.0)^a^	100 (±0.0)^a^
Permethrin 2.5 g/L spray	18 (±2.1)^d^	14 (±0.8)^d^	21(±2.6)^d^	25 (±1.1)^c^
DEET-1	8 (±1.8)^de^	0^e^	12 (±2.5)^d^	0^d^
Negative control	0^e^	0^e^	0^e^	0^d^

^a^Treatment with ca. two-fold LD_90_ value of each sample; Exposed for 24 h. ^b^Each experiment was performed 3 times and the data averaged; Means within a column followed by the same letter are not significantly different (*P* = 0.05, Scheffé test). ^c^A, direct application method; B, indirect application method.

## Discussion

Although many investigations have explored the potential plant-derived acaricides for house dust mite control, their major limitation is that allergen-containing dead mites were not removed. Hence, in the present study, we focused on developing a mite indicator to completely remove the house dust mites from the affected area. The results of this study showed variation in the acaricidal activities of the three oils of *M*. *officinalis* cultivated in France, Ireland, and Serbia with respect to geographical regions. *M*. *officinalis* oil from France was more active against *D*. *farinae* and *D*. *pteronyssinus*, compared to *M*. *officinalis* oil cultivated in Ireland and Serbia. An interesting result was the color deformation effect of *M*. *officinalis* oils cultivated in France, Ireland, and Serbia against *D*. *farinae* and *D*. *pteronyssinus*. Our results confirm that the bodies of house dust mites were obviously changed to golden brown from colorless, after treatment with the three oils of *M*. *officinalis* cultivated in France, Ireland, and Serbia (Fig. 1). In addition, the color alteration of the mites treated with *M*. *officinalis* oil allowed *D*. *farinae* and *D*. *pteronyssinus* to be distinguished with the naked eye. This reaction could be caused by polyphenol oxidase (PPO) and tyrosinase^15^. The PPO exists in propolyphenol oxidase form in both insects and house dust mites, and is involved in immunity and self-recognition^15^. It is thought that disease resistance occurs due to the existence of polyphenol oxidase^16^. Furthermore, tyrosinase is an oxidase that controls the production of melanin in plants and animals^15^.

The three oils of *M*. *officinalis* cultivated in France, Ireland, and Serbia exhibited a number of common main components in variable compositions. The oil of *M*. *officinalis* cultivated in France was rich in 3,7-dimethyl-2,6-octadienal (43.37%), but the samples cultivated in Ireland and Serbia contained more β-caryophyllene (30.75 and 32.71%, respectively) than 3,7-dimethyl-2,6-octadienal (26.90 and 21.88%, respectively). These changes in the compositions of the essential oils may be influenced by several environmental (climatic and seasonal variation, geographical origin) and genetic differences^17,18^. Previous studies reported that the compositions of the essential oils were significantly dependent upon the locations, including altitude, where the plants grew^19,20^. Moreover, many researchers have reported the variation in chemical compositions of the plant essential oils with respect to geographical origin^20–23^. Consequently, acaricidal activities were dependent upon the geographical origin of the essential oils, since the main compounds of the essential oils determine their bioactivities^18^.

The superior acaricidal and color deformation potential of *M*. *officinalis* oil cultivated in France could be attributed to the high amount of the main components. In our study, the acaricidal and color deformation principle of *M*. *officinalis* oil (France) was identified as 3,7-dimethyl-2,6-octadienal. Of the major constituents tested, high toxicity was obtained from caryophyllene oxide, β-caryophyllene, 6-methyl-5-hepten-2-one, 3,7-dimethyl-6-octenal, and 3,7-dimethyl-2,6-octadienal against *D*. *farinae* and *D*. *pteronyssinus*. The acaricidal toxicity of geranyl acetate is comparable with that of DEET. On the other hand, the color alteration of mite bodies was only observed when the *D*. *farinae* and *D*. *pteronyssinus* were treated with 3,7-dimethyl-2,6-octadienal. In our previous studies on benzaldehyde derivatives from *Morinda officinalis*, 2,3-dihydroxybenzaldehyde was found to be toxic to *Dermatophagoides* spp., and caused color alteration to a dark brown color of the body^24^. The *M*. *officinalis* oil and 3,7-dimethyl-2,6-octadienal have advantages over 2,3-dihydroxybenzaldehyde, because of their safety for humans. *M*. *officinalis* oil and 3,7-dimethyl-2,6-octadienal are on the FDA’s generally recognized as safe (GRAS) list. Furthermore, 3,7-dimethyl-2,6-octadienal has been approved as a food additive by the European Commission (EC), because its use does not pose a risk to consumers’ health status^25^. Fukumoto *et al*.^26^ reported that physical and psychological stress may be alleviated by the ingestion of lemon oil containing constituents such as limonene and 3,7-dimethyl-2,6-octadienal. According to Kennedy *et al*.^27^, the negative effects of the Defined Intensity Stressor Simulation (DISS) were ameliorated by acute administration of *M*. *officinalis* oil (600 mg dose), with considerable increase in “calmness” and reduced “alertness”. Taking into account their acaricidal properties, color deformation effects, pleasant fruity scent, and safety for humans, *M*. *officinalis* oil (France) and 3,7-dimethyl-2,6-octadienal might become a suitable acaricidal and mite indicator ingredient for the control of *D*. *farinae* and *D*. *pteronyssinus*.

**Figure 3:**
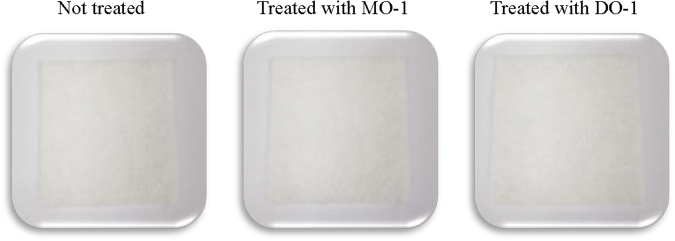
Staining effect of spray formulations on white cotton. Each test sample was then sprayed two times successively at 15 cm upwards.

Investigations of the toxic action mechanisms of naturally occurring acaricides are of practical importance for house dust mite control, because they may provide valuable information on the most suitable formulations to be adopted for their future commercialization^28^. In the present study, *M*. *officinalis* oil (France) and 3,7-dimethyl-2,6-octadienal were significantly more effective in closed containers than in open containers. These results indicate that the acaricidal route of action of these compounds was largely a result of vapor action, although the exact mechanism of the oil remains unknown. The fumigant action of *M*. *officinalis* oil (France) and 3,7-dimethyl-2,6-octadienal described, as demonstrated through the current fumigant chamber test (Table 4), is of practical importance, because it allows the essential oil or its main compound to reach deep refugees in blanket, pillows, carpet, and other fabric materials. This fumigant action system has advantages over the contact action system, because exposure to active constituents can be easily controlled in a closed space using suitable application methods^29^. In our study, direct and indirect application of sprays containing 0.5 or 1% *M*. *officinalis* oil (France) and 3,7-dimethyl-2,6-octadienal to *D*. *farinae* and *D*. *pteronyssinus* adults gave rapid action and excellent toxicity. These sprays produced more than 80% mortality against *D*. *farinae* and *D*. *pteronyssinus* adults 4 h after treatment. Complete mortalities and color alteration of mites were achieved using 0.5% and 1% sprays containing 3,7-dimethyl-2,6-octadienal and 1% spray containing *M*. *officinalis* oil (France) (Supplementary Fig. S2). No staining was observed in white cotton treated with 1% *M*. *officinalis* oil (France) and 3,7-dimethyl-2,6-octadienal (Fig. 3). Thus, such spray formulations have great advantage, especially in situations where staining is an issue, such as carpet, mattress, and pillow. Because of the high volatility of the *M*. *officinalis* oil (France) and 3,7-dimethyl-2,6-octadienal described, the binary mixture formulations of *M*. *officinalis* oil (France) or 3,7-dimethyl-2,6-octadienal and acaricidal compounds with contact action (e.g. 3-methylacetophenone^30^, or 4-chloro-6-isopropyl-3-methylphenol^31^) could be useful agent for mite control in space where a window is open.

In conclusion, certain plant essential oils and their constituents, such as *M*. *officinalis* oil (France) and 3,7-dimethyl-2,6-octadienal, may be best used as safe control agents and mite indicators against *D*. *farinae* and *D*. *pteronyssinus*. For the practical use of *M*. *officinalis* oil (France) and 3,7-dimethyl-2,6-octadienal as novel acaricides and mite indicators to proceed, further investigation is necessary on the development of formulations (aerosol, smoking agent, or fumigant) to improve acaricidal efficacy and stability.

## Materials and Methods

### Chemicals

Caryophyllene oxide (95%), β-caryophyllene (98.5%), geranyl acetate (97%), 6-methyl-5-hepten-2-one (98%), octanal (99%), 2-octenal (95%), 2,4-octadienal (95%), 3,7-dimethyl-6-octenal (95%), 3,7-dimethyl-2,6-octadienal (96%), 3,7-dimethyl-1-octene (97%), and 3,7-dimethyl-1,3,6-octatrien (90%) were purchased from Aldrich (Missouri, USA). 3,7-Dimethyl-1,6-octadiene (98%) was provided by Sigma-Aldrich (Dorset, UK), and DEET (95%) was supplied by Fluka (Buchs, Switzerland). Permethrin (*cis:trans*, 25:75) 2.5 g/L was purchased from Avention Co. Ltd (Incheon, South Korea). Ethoxylated castor oil (emulsifier), was a gift from the Special Fine Chemical (SFC) Co. Ltd. (Yeosu, South Korea).

### Plant material and isolation of essential oil

Leaves of *M*. *officinalis* (*n* = 3) cultivated in France, Ireland, and Serbia were purchased from a local market in Jeonju, South Korea in May 2017. The samples were extracted using the steam distillation extraction technique. The water was removed on anhydrous magnesium sulfate, and the extracted oil was concentrated to dryness by rotary evaporation at 26 °C. The essential oil was kept at 4 °C to prevent volatile compounds.

### House dust mites

The cultures of *D*. *farinae* and *D*. *pteronyssinus* were separately maintained without exposure to any known acaricide in the laboratory for more than 10 years. The mites were reared in mite rearing chamber (see Supplementary Fig. S3).

### Gas chromatography-mass spectrometry (GC-MS)

The essential oils were analyzed on a GC-MS (HP 6890 and 5973 IV, Agilent Technologies, Palo Alto, USA). The GC column was a DB-5 (0.25 mm film) fused silica capillary column (30 m × 0.25 mm i.d. × 0.25 μm thickness). The GC oven temperature was programmed from 51 °C to 211 °C, then increased to 200 °C at 2 °C/min, and held at this temperature for 15 min. Helium was used as the carrier gas at a rate of 0.81 mL/min for the analysis of the essential oils. The essential oil was introduced directly into the MS. Mass spectra were obtained by automatic scanning in the mass range *m/z* 50–600 for 2 seconds. Chromatographic peaks confirmed the retention index, retention time, and mass spectra, by comparison with the published mass spectra data^32^.

### Preparation of spray formulations

Three spray formulations containing *M*. *officinalis* oil (France) and 3,7-dimethyl-2,6-octadienal, respectively, in 5 mL plastic containers with a pump spray nozzle (Uncleg, Hwaseong) were prepared, to determine the effective acaricidal products (Supplementary Table S2) for the control of *Dermatophagoides* spp. Single spray applications of 0.25, 0.5, and 1% concentrations of the *M*. *officinalis* oil and 3,7-dimethyl-2,6-octadienal preparations delivered ca. 2.11, 4.22 and 8.44 µg/cm^2^ of total material to a filter paper (5.5 cm i.d. × 25 μm thickness, Whatman, Maidstone, UK), respectively.

### Contact + fumigant mortality bioassay

A contact + fumigant mortality bioassay was modified from the method described by Yun *et al*.^28^ Various concentrations (104, 52, 26, 19.5, 13, 6.5, 3.25, 1.63 and 0.82 µg/cm^2^) of the three *M*. *officinalis* oils (France, Ireland, and Serbia) and all compounds were dissolved in ethanol (100 µL), and applied to 5.5 cm diameter filter paper. After drying under a fume hood for 2 min, each filter paper was placed in the bottom section of a petri dish (5.5 cm i.d. × 1.5 cm deep), and then 30 randomly selected adult mites (both sexes, 8–10 days old) of *D*. *farinae* and *D*. *pteronyssinus* were inoculated in each petri dish, and the lid was sealed. Positive control with the *N*,*N*-diethyl-m-toluamide, a commonly used acaricide for mite control, was similarly formulated. Negative controls received ethanol (100 µL) only.

The treated and control mites were maintained for 24 h at 24 °C and 74% relative humidity in darkness. The mortality of each bioassay was determined by observing the number of mites under a binocular microscope (40×, Olympus, Tokyo, Japan). All experiments were replicated three times.

### Color deformation effects

Color deformation effects of *M*. *officinalis* oil cultivated in France, Ireland and Serbia and their major commercial components against *D*. *farinae* and *D*. *pteronyssinus* were investigated by the methods of Lee *et al*.^4^. Approximately two-fold concentrations of the contact + fumigant LD_90_ values of each test sample were applied to 5.5 cm diameter filter paper. After drying under a fume hood for 2 min, each filter paper was placed in the bottom section of a petri dish (5.5 cm i.d. × 1.5 cm deep), and then 80–100 randomly selected adult mites (both sexes, 8–10 days old) of *D*. *farinae* and *D*. *pteronyssinus* were inoculated in each petri dish, and the lid was sealed as stated in above (contact + fumigant mortality bioassay section). A color alteration of the mites was compared with color deformation before and after treatment and conducted using a light microscope (40× and 100×) and naked eye.

### Vapor phase mortality bioassay

The closed and open container method, described by Kwon and Ahn^29^, was used to determine whether the lethality of *M*. *officinalis* oil (France) and 3,7-dimethyl-2,6-octadienal against adults of *D*. *farinae* and *D*. *pteronyssinus* was attributable to contact or fumigant (vapor) action. A group of 30 randomly selected adult mites (both sexes, 8–10 days old) were introduced into untreated 5.5 cm diameter filter paper on the bottom of a petri dish (5.5 cm i.d. × 1.5 cm deep), and each petri dish was covered with a fine cotton mesh. Approximately two-fold concentrations of the contact + fumigant LD_90_ values of each test material were applied to 5.5 cm diameter filter papers, as stated in contact + fumigant mortality bioassay. Each treated filter paper was separately placed on top of the cotton mesh (6.5 cm diameter), to prevent direct contact of mites with the test sample. Each petri dish was sealed either with another tight-fitting lid (closed container method), or with another tight-fitting lid with a 3 cm central hole (open container method), to determine the potential vapor phase toxicity of the test samples. Negative controls received ethanol (100 µL) only.

The treated and control mites were maintained for 24 h at 24 °C and 74% relative humidity in darkness. The mortality of each bioassay was determined by observing the number of mites under a binocular microscope (20×, Olympus, Tokyo, Japan). All experiments were replicated three times.

### Bioassay for fumigant action

Fumigant chambers covered with non-woven fabric of three thicknesses (10, 25, and 30 mm) were used to determine whether the *M*. *officinalis* oil (France) and 3,7-dimethyl-2,6-octadienal were effective in the control of *D*. *farinae* and *D*. *pteronyssinus* that live deep in pillows, carpet, and other fabric materials. A fumigation chamber was constructed with two petri dish bottoms (5.5 cm i.d. × 1.5 cm deep each), stacked lip-to-lip to create a total volume of 71.24 cm^3^ (Supplementary Fig. S1). The two plastic petri dish bottoms were separated by a 7 × 7 cm section of non-woven fabric of the three thicknesses. Approximately two-fold concentrations of the contact + fumigant LD_90_ values of each test samples were applied to 5.5 cm diameter filter. After drying under a fume hood for 3 min, each treated filter paper was attached with double-sided adhesive tape (3 M, South Korea) to the lid of the upper chamber. For each test, 30 adult mites (both sexes, 8–10 days old) were introduced into the untreated 5.5 cm diameter filter paper on the bottom of the lower chamber. A wad of cotton saturated with distilled water (50 µL) had been introduced to the bottom of the lower chamber. The two plastic petri dish bottoms were held together, and wrapped in several layers of Parafilm^®^, and the mites were held at 24 °C and 74% relative humidity for 24 h. The mortality of each bioassay was determined by observing the number of mites under a binocular microscope (20×, Olympus, Tokyo, Japan). All experiments were replicated three times.

### Spray bioassay

Direct and indirect spray application methods described by Kim *et al*.^33^ and Yun *et al*.^28^ were used to investigate the efficacy of the three spray formulations against adult *D*. *farinae* and *D*. *pteronyssinus*. For the direct spray application method, groups of 40 adult mites (both sexes, 8–10 days old) were placed on 5.5 cm diameter filter paper 5 min prior to spraying. Each test sample was then sprayed two times successively at 15 cm upwards onto the 5.5 cm diameter filter paper. For the indirect spray application method, each test sample was sprayed two times successively at 15 cm upwards onto the filter paper (5.5 diameter). Groups of 30 randomly selected adult mites (both sexes, 8–10 days old) were introduced into treated 5.5 cm diameter filter paper on the bottom of a petri dish (5.5 cm i.d. × 1.5 cm deep), and each petri dish was sealed with lid. For comparison, DEET and permethrin (*cis:trans* 25:75) 2.5 g /L were served as the positive controls. Because permethrin (*cis:trans* 25:75) 2.5 g /L spray has been approved by the Ministry of Food and Drug Safety (MFDS, South Korea), the commercial acaricide served as positive controls for spray bioassays. Ethoxylated castor oil (surfactant) + ethanol + distilled water was served as the negative control. The treated and control mites were maintained for 24 h at 24 °C and 74% relative humidity in darkness. The mortality of each bioassay was determined by observing the number of mites under a binocular microscope (40× and 100×, Olympus, Tokyo, Japan). All experiments were replicated three times.

### Data analysis

The control mortality was corrected by Abbott’s formula^34^. The 50 and 90% lethal dose (LD_50_ and LD_90_) values were calculated by probit analysis^35^. Mortality percentages were transformed to arcsine square root values for analysis of variance (ANOVA). Student’s t-test was used to test for significant differences between the closed container and open container methods. The Scheffé test was used to test for significant differences among the treatments. Means (±SE) of untransformed data were reported.

## Electronic supplementary material

Supplementary info
