# Supplementary material for: Acaricidal target and mite indicator as color alteration using 3,7-dimethyl-2,6-octadienal and its derivatives derived from Melissa officinalis leaves
Source: Sci Rep. 2018 May 25;8:8129. doi: 10.1038/s41598-018-26536-9 (PMC5970210; doi:10.1038/s41598-018-26536-9)
Supplement: Supplementary file 1 — Supplementary info [file 41598_2018_26536_MOESM1_ESM.pdf]

Acaricidal target and mite indicator as color alteration using 3,7-dimethyl-2,6-octadienal and its derivatives derived from *Melissa officinalis* leaves

Jun-Hwan Park[1], Hoi−Seon Lee[1]

[1]Department of Bioenvironmental Chemistry, Chonbuk National University, Jeonju 54896, Republic of Korea

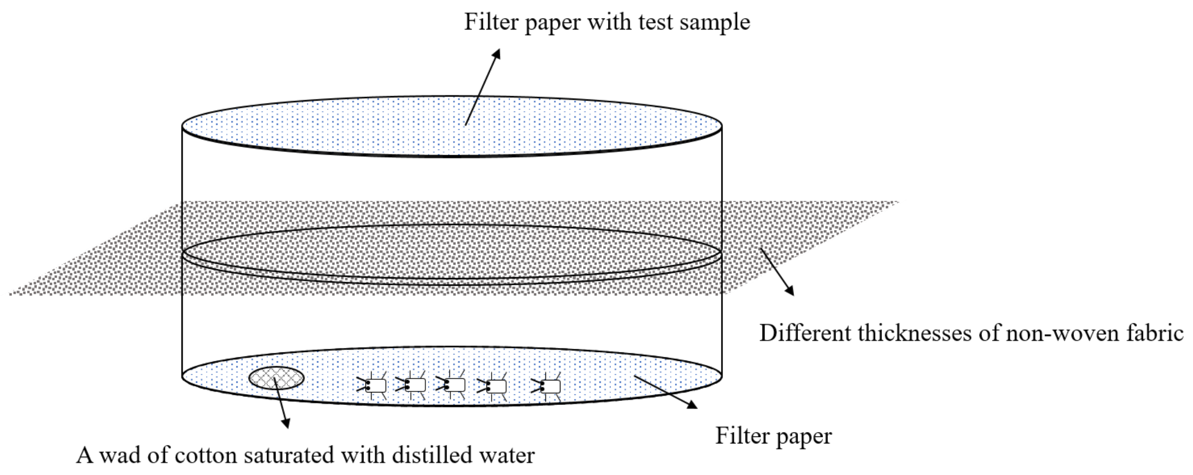

Supplementary Figure S1. Fumigation chamber consists of 2 plastic petri dish bottoms (6.0 cm in diameter by 1.5 cm in height and a total volume 71.24 cm^3.

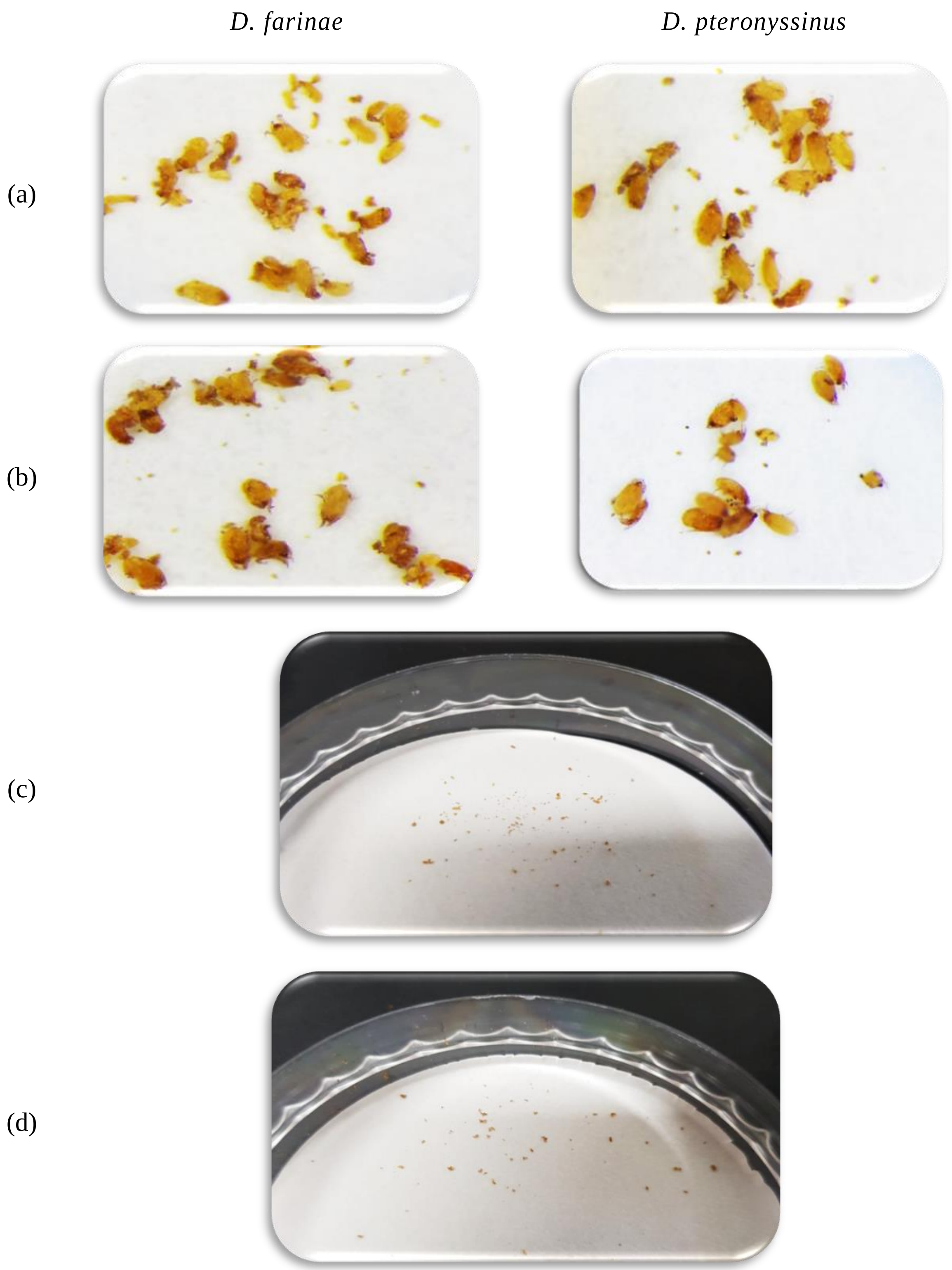

Supplementary Figure S2. Color deformation effects of spray formulations against American house dust mites and European house dust mites, using indirect application methods. (a) mites, treated with MO-1, (b) mites, treated with DO-1 (40×), (c) mites, treated with MO-1 (naked eye), and (d) mites, treated with DO-1 (naked eye).

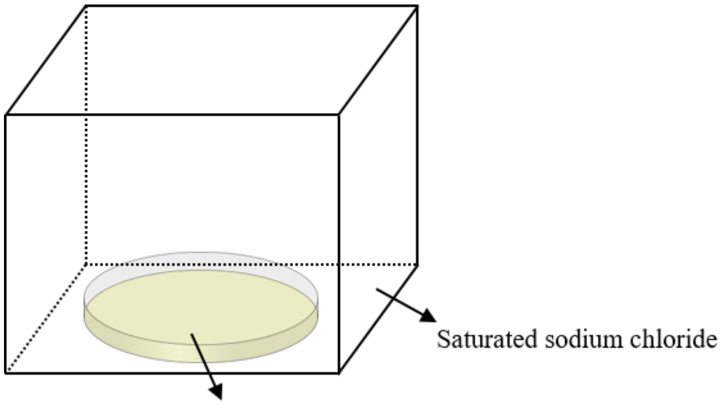

Supplementary Figure S3. The mites were reared in plastic circle container (10 cm i.d. × 4.0 cm deep) containing 45 g of fiy feed (consist of protein, cellulose, lipid, phosphorus, calcium, and others) + dried yeast. The petri dish was placed in the bottom section of rearing chamber (17 × 17 × 15 cm). The saturated sodium chloride (20 ml) were applied to bottom of rearing chamber to prevent escape of the mites. Rearing chambers were kept in an incubator at 26 ± 2 ℃ and 74% relative humidity in dark.

Supplementary Table S1. Relative composition of the essential oils of *M. officinalis* cultivated in France, Ireland, and Serbia. ([a]Retention index, Kovats index of retention)

Compound	Retention Time	RI[a]	Relative composition (%)			Molecular mass (g/mol)	Molecular formula
			France	Ireland	Serbia		
6-Methyl-5-hepten-2-one	5.83	938	1.53	1.10	-	126.20	$C_8H_{14}O$
3,7-Dimethyl-1,3,6-octatriene	6.99	976	1.38	1.61	2.81	136.24	$C_{10}H_{16}$
3,7-Dimethyl-6-octenal	8.89	1125	1.95	8.65	7.67	154.25	$C_{10}H_{18}O$
3,7-Dimethyl-2,6-octadienal	10.43	1174	43.37	26.90	21.88	152.24	$C_{10}H_{16}O$
Methyl citronellate	10.65	1203	0.35	2.79	2.06	184.28	$C_{11}H_{20}O_2$
Geranyl acetate	12.51	1352	2.10	2.27	0.46	196.29	$C_{12}H_{20}O_2$
α-Copaene	12.57	1211	0.84	2.01	1.70	204.36	$C_{15}H_{24}$
β-Elemene	12.77	1398	0.76	0.85	1.33	204.36	$C_{15}H_{24}$
β-Caryophyllene	13.28	1415	27.42	30.75	32.71	204.36	$C_{15}H_{24}$
Germacrene D	14.11	1478	14.46	-	-	204.36	$C_{15}H_{24}$
α-Farnesene	14.26	1458	0.92	-	1.76	204.36	$C_{15}H_{24}$
δ-Cadinene	14.59	1469	1.70	7.97	-	204.36	$C_{15}H_{24}$

β-Cadinene	14.84	1508	-	-	3.90	204.36	$C_{15}H_{24}$
Caryophyllene oxide	15.48	1507	0.89	-	-	220.36	$C_{15}H_{24}O$
β-Cubebene	16.23	1345	-	11.19	21.54	204.36	$C_{15}H_{24}$
**Major grouped compounds**							
Monoterpene hydrocarbon			1.38	1.61	2.81		
Monoterpene alcohol			1.53	1.10	-		
Monoterpene aldehyde			45.32	35.55	29.55		
Monoterpene ester			2.45	5.06	2.52		
Sesquiterpene hydrocarbons			46.99	52.77	62.94		
**Total**			97.67	96.09	97.82		

Supplementary Table S2. Three experimental spray formulations containing *M. officinalis* oil from France and 3,7-dimethyl-2,6-octadienal.

Spray formulation	Percentage content (%)					
	M. officinalis oil	3,7-dimethyl-2,6-octadienal	DEET	Castor oil[a]	Ethanol	Distilled water
MO-0.25[b]	0.25	-	-	0.25	10	89.50
MO-0.5	0.5	-	-	0.25	10	89.25
MO-1	1	-	-	0.25	10	88.75
DO-0.25[c]	-	0.25	-	0.25	10	89.50
DO-0.5	-	0.5	-	0.25	10	89.25
DO-1	-	1	-	0.25	10	88.75
DEET-1[d]	-	-	1	0.25	10	88.75
Control	-	-	-	0.25	10	89.75

[a]Ethoxylated castor oil.

[b]*M. officinalis* oil 0.25, 0.5 and 1%

[c]3,7-dimethyl-2,6-octadienal 0.25, 0.5 and 1%.

[d]DEET 1%.
